# The Bacteriophage EF-P29 Efficiently Protects against Lethal Vancomycin-Resistant *Enterococcus faecalis* and Alleviates Gut Microbiota Imbalance in a Murine Bacteremia Model

**DOI:** 10.3389/fmicb.2017.00837

**Published:** 2017-05-09

**Authors:** Mengjun Cheng, Jiaming Liang, Yufeng Zhang, Liyuan Hu, Pengjuan Gong, Ruopeng Cai, Lei Zhang, Hao Zhang, Jinli Ge, Yalu Ji, Zhimin Guo, Xin Feng, Changjiang Sun, Yongjun Yang, Liancheng Lei, Wenyu Han, Jingmin Gu

**Affiliations:** ^1^Key Laboratory of Zoonosis Research, Ministry of Education, College of Veterinary Medicine, Jilin UniversityChangchun, China; ^2^College of Clinical Medicine, Jilin UniversityChangchun, China; ^3^First Hospital of Jilin University, Jilin UniversityChangchun, China; ^4^Jiangsu Co-innovation Center for the Prevention and Control of important Animal Infectious Disease and ZoonosesYangzhou, China

**Keywords:** vancomycin-resistant *Enterococcus faecalis*, phage therapy, bacteremia, gut microbiota, opportunistic pathogens

## Abstract

*Enterococcus faecalis* is becoming an increasingly important opportunistic pathogen worldwide, especially because it can cause life-threatening nosocomial infections. Treating *E. faecalis* infections has become increasingly difficult because of the prevalence of multidrug-resistant *E. faecalis* strains. Because bacteriophages show specificity for their bacterial hosts, there has been a growth in interest in using phage therapies to combat the rising incidence of multidrug-resistant bacterial infections. In this study, we isolated a new lytic phage, EF-P29, which showed high efficiency and a broad host range against *E. faecalis* strains, including vancomycin-resistant strains. The EF-P29 genome contains 58,984 bp (39.97% G+C), including 101 open reading frames, and lacks known putative virulence factors, integration-related proteins or antibiotic resistance determinants. In murine experiments, the administration of a single intraperitoneal injection of EF-P29 (4 × 10^5^ PFU) at 1 h after challenge was sufficient to protect all mice against bacteremia caused by infection with a vancomycin-resistant *E. faecalis* strain (2 × 10^9^ CFU/mouse). *E. faecalis* colony counts were more quickly eliminated in the blood of EF-P29-protected mice than in unprotected mice. We also found that exogenous *E. faecalis* challenge resulted in enrichment of members of the genus *Enterococcus* (family *Enterococcaceae*) in the guts of the mice, suggesting that it can enter the gut and colonize there. The phage EF-P29 reduced the number of colonies of genus *Enterococcus* and alleviated the gut microbiota imbalance that was caused by *E. faecalis* challenge. These data indicate that the phage EF-P29 shows great potential as a therapeutic treatment for systemic VREF infection. Thus, phage therapies that are aimed at treating opportunistic pathogens are also feasible. The dose of phage should be controlled and used at the appropriate level to avoid causing imbalance in the gut microbiota.

## Introduction

*Enterococcus faecalis* is a Gram-positive bacterium found in plants and the soil and acts as a commensal in the gastrointestinal tract of humans, mammals, and insects (Guzman Prieto et al., 2016). Despite its commensal nature, this bacterium has also become a globally important nosocomial pathogen that can cause bacteremia, endocarditis, intra-abdominal, pelvic, urinary tract, skin and skin structure infections, and, rarely, central nervous system infections, in immune-compromised patients (Murray, 1990; Jett et al., 1994). *E. faecalis* exhibits intrinsic resistance to a broad range of antibiotics (Guzman Prieto et al., 2016). In recent years, the treatment of infections caused by *E. faecalis* has become increasingly difficult because of the prevalence of multidrug-resistant *E. faecalis* strains, especially vancomycin-resistant strains, and this has raised serious concerns within the medical community (Emaneini et al., 2016).

Bacteriophages (phages) are bacterial viruses that specifically infect bacteria, hijack their machinery, replicate intracellularly, and then finally lyse the host bacterium before being released by the host cell (Wittebole et al., 2014). Although phage therapy was explored as an antimicrobial treatment for bacterial infectious diseases in the early 1920s, the development of phage therapy was slowed by the advent of antibiotics (Sulakvelidze et al., 2001). Recently, the rise of antibiotic-resistant bacteria has led to an increase in interest in phage therapy (Nobrega et al., 2015). Phage therapy might be a viable alternative to or complement for conventional antibiotic therapy because it has already been proven to be advantageous and more specific, accurate and potent than antibiotics based on the *in vitro* and animal models studies (Gu et al., 2012). However, key regulatory barriers continue to prevent the approval of phage in human therapies by the US FDA (Food and Drug Administration) and the EMA (European Medicines Agency) (Keen, 2012; Cooper et al., 2016). So, phage therapy need a more comprehensive and detailed study.

Several studies have explored the effects of *E. faecalis* phage administration *in vivo* (Biswas et al., 2002; Uchiyama et al., 2008; Letkiewicz et al., 2009; Zhang et al., 2013; Duerkop et al., 2016). However, it has been reported that there exist more than 10^31^ phage particles in the biosphere (Hatfull and Hendrix, 2011). Considering the diversity of phages, this sampling is only “the tip of the iceberg” (Rohwer, 2003). More sampling from different ecological regions is necessary. Hence, EF-P29, a novel *E. faecalis* phage, was isolated and characterized in this study. Additionally, there are no reports showing whether *E. faecalis* phage administration affects the commensal *E. faecalis* that colonizes the gastrointestinal tract of humans and other mammals. Therefore, the treatment effect of EF-P29 against lethal vancomycin-resistant *E. faecalis* in a murine bacteremia model and the influence of this phage on the gut microbiota were studied.

## Materials and Methods

### Isolation and Identification of *E. faecalis*

*Enterococcus faecalis* was isolated from clinical specimens obtained from patients at the First Hospital of Jilin University (Changchun, Jilin province, China). All of the isolated strains were confirmed to be *E. faecalis* using 16S rRNA and SodA sequence analysis after PCR amplification with the universal primers E1 (5′-TCAACCGGGGAGGGT-3′) and E2 (5′-ATTACTAGCGATTCCGG-3′), and FL1 (5′-ACTTATGTGACTAACTTAACC-3′) and FL2 (5′-TAATGGTGAATCTTGGTTTGG-3′) (Layton et al., 2010). *E. faecalis, Enterococcus faecium, Staphylococcus aureus*, and *Streptococcus* species were cultured in Brain Heart Infusion (BHI) broth (tryptone 10 g/l; bovine brain leaching powder 12.5 g/l; bovine heart leaching powder 5 g/l; NaCl 5 g/l; glucose 2 g/l; Na_2_HPO_3_⋅12⋅H_2_O 2.5 g/l. Becton, Dickinson and Company, USA), whereas other species (including *Bacillus subtilis, Escherichia coli, Pseudomonas aeruginosa*, and *Klebsiella pneumoniae*) were cultured in Luria-Bertani (LB) medium (tryptone 10 g/l; NaCl 10g/l; yeast 5 g/l. Becton, Dickinson and Company, USA). All bacterial strains were stored in 30% glycerol at -80°C and routinely cultivated at 37°C.

Bacterial genomic DNA samples obtained from *E. faecalis* isolates were amplified using randomly amplified polymorphic DNA-analysis (RAPD-PCR), as previously described (Yang et al., 2015). The oligonucleotide that was used in the RAPD-PCR was 5′-AACGCGCAAC-3′ (Martin et al., 2005). The RAPD products were then separated using electrophoresis and analyzed using BioNumerics (Applied Maths, St. Martens-Latem, Belgium).

The antibiotic susceptibility of *E. faecalis* isolates was evaluated using the Kirby-Bauer disk diffusion method according to the criteria of the Clinical and Laboratory Standards Institute (Performance standards for antimicrobial susceptibility testing. M100–S20 CLSI, Wayne, PA, USA, 2010). Antibiotics that are commonly used in the hospital were selected and bought from the Hangzhou Binhe Microorganism Reagent Development Company, Hangzhou, China, including vancomycin (30 μg), erythromycin (15 μg), streptomycin (300 μg), chloromycetin (30 μg), ampicillin (30 μg), gentamicin (120 μg), tetracycline (30 μg), nitrofurantoin (300 μg), ciprofloxacin (5 μg), rifampicin (5 μg), fosfomycin (200 μg), daptomycin (15 μg), linezolid (30 μg).

### Isolation and Characteristics of Phage

The *E. faecalis* strain GF29 was used to isolate phages from sewage samples that were collected from the Changchun sewerage system (Gong et al., 2016). Phages were detected and purified using the double-layer agar plate method (Gu et al., 2011). The phages were amplified and stored at either 4°C or at -80°C in glycerol (3:1 [vol/vol]).

The concentration and purification of the phages was conducted as previously described with some modifications (Matsuzaki et al., 2003). After we performed large-scale culturing to grow the phage EF-P29, the lysates were centrifuged at 8,000 × *g* for 10 min at 4°C to remove cell debris. The phage particles were precipitated from the culture supernatants using 10% polyethylene glycol 8,000 and 1 M NaCl and then dissolved in 5 ml phosphate-buffered saline (PBS; 137 mM NaCl, 2.7 mM KCl, 50 mM Na_2_HPO_4_ and 10 mM KH_2_PO_4_, pH 7.4). The phage suspension was placed on top of a discontinuous CsCl gradient (1.45, 1.50, and 1.70 g/ml) and centrifuged at 35,000 × *g* for 3.5 h at 4°C. The phage band was then collected and dialyzed. The concentrated EF-P29 samples were placed on carbon-coated copper grids and allowed to absorb for 15 min. They were then negatively stained with phosphotungstic acid (PTA, 2% w/v). The morphology of EF-P29 was examined using a transmission electron microscope (TEM) (JEOL JEM-1200EXII, Japan Electronics and Optics Laboratory, Tokyo, Japan) at an acceleration voltage of 80 kV.

The host spectrum of the phage EF-P29 was characterized using spot testing (Chang et al., 2005) and the double-layer agar plate method (Gu et al., 2011), as previously described, with some modifications.

The multiplicity of infection (MOI) was defined as the ratio of virus particles to host cells (Gu et al., 2011). The *E. faecalis* strain GF29 was cultivated to log phase in BHI broth (OD_600_ = 0.6) and infected with the phage EF-P29 at different MOIs (0.001, 0.01, 0.1, 1, 10, and 100). After the cultures were incubated while shaking for 8 h at 37°C, the titers of the lysates were quantified (Gu et al., 2011).

The one-step growth curve of the phage EF-P29 was determined as previously described, with some modifications (Pajunen et al., 2000). A culture of GF29 was grown to mid-exponential phase and then harvested and resuspended in fresh BHI broth. Phages were added to mid-exponential phase cultures of GF29 at an MOI of 0.01 and allowed to adsorb for 10 min at 37°C. The mixture was centrifuged at 4°C for 10 min at a speed of 10,000 × *g*, and the pellets were then resuspended in 10 ml of BHI. This suspension was incubated at 37°C while shaking. Samples of the mixture were obtained at 10-min intervals, immediately diluted, and then plated for phage titration.

### Sequencing and Bioinformatics Analysis of the EF-P29 Genome

Phage genomic DNA was extracted from concentrated and purified EF-P29 preparations using a viral genome extraction kit (Omega Bio-Tek Inc., Norcross, GA, USA). Whole genome sequencing was performed by Suzhou GENEWIZ Biotechnology Co. Ltd using IlluminaHiSeq 2500 sequencing. The bioinformatics analysis of the complete genome sequence of the phage EF-P29 was performed as previously described (Gong et al., 2016). The circle map of the EF-P29 genome was constructed using CGView^1^ (Grant and Stothard, 2008). Coding sequences (CDSs) and potential open reading frames (ORFs) were predicted and analyzed using BLAST and GeneMarkS (Besemer et al., 2001). Genes encoding tRNA were identified by tRNAscan-SE (Lowe and Eddy, 1997). The gene and deduced amino acid sequences were used to perform database searches (using standard parameters) in BLASTN and BLASTP, respectively, using the National Center for Biotechnology Information (NCBI) network service (Altschul et al., 1997).

### Inhibitory Effect of the Phage EF-P29 on Vancomycin-Resistant *E. faecalis In Vitro*

The ability of EF-P29 to inhibit the growth of the vancomycin-resistant *E. faecalis* (VREF) strain 002 was analyzed using colony counting. Briefly, a culture of the VREF 002 strain was grown to mid-exponential phase, harvested and resuspended in fresh BHI broth. The VREF 002 strain and previously prepared EF-P29 dilutions in PBS buffer were inoculated in 2× BHI. The mixture was diluted to make 1× BHI and then normalized to reach a final bacterial count of 10^8^ CFU/ml. The phages were added at an MOI of 0.0001, 0.001, 0.01, 0.1, or 1 in separate tubes. Consequently, the *in vitro* lytic efficiency of the phages could be examined. Bacterial growth in the absence of phages was analyzed as the control. The tubes were incubated at 37°C for 8 h while shaking, and the bacterial density of the cultures was measured. Each independent trial was repeated three times.

### Mouse Experiments

Female BALB/c mice aged 6–8 weeks old (weight, 18 to 20 g) were purchased from the Experimental Animal Center of Jilin University, Changchun, China. Water and normal mouse chow were provided *ad libitum*, and the mice were monitored daily. Groups of five mice per experiment were intraperitoneally (i.p.) injected with different inocula of the VREF strain 002 (1 × 10^6^, 1 × 10^7^, 1 × 10^8^, 1 × 10^9^, and 1 × 10^10^ CFU/mouse) to determine the minimal dose that would produce 100% mortality over a 7-day follow-up period (the minimal lethal dose [MLD]) (Zhang et al., 2013). Once the MLD was determined, 2× MLD was used as the infective inoculum (the challenge dose).

To determine the effect of the phage treatment in mice, a single dose of EF-P29 (4 × 10^3^, 4 × 10^4^, 4 × 10^5^, 4 × 10^6^, or 4 × 10^7^ PFU/mouse) was administered i.p. 1 h after bacterial challenge. Each dose group contained 10 mice. In the control group, the infected mice were treated with PBS buffer. The ability of EF-P29 to protect the bacteremic mice was investigated using colony counting. At predetermined intervals, bacterial counts were determined in 10 μl of peripheral blood obtained from the caudal veins of mice that were treated with either EF-P29 or buffer.

To verify the effect of phage therapy on the health of the mice, body weight was measured initially, weekly and at the end of the experiment in all of the mice (Chang et al., 2015; Wang et al., 2016). Health scores were determined using a method that was modified from a previous report (Xia et al., 2015). The mice were scored for their state of health on a scale of 5 to 0 based on disease progression at 24 h after treatment with a specific therapy. A score of 5 indicates normal health and an unremarkable condition. Slight illness was defined as decreased physical activity and ruffled fur and was scored as 4. Moderate illness was defined as lethargy and a hunched back and was scored as 3. Severe illness was defined as the aforementioned signs plus exudative accumulation around partially closed eyes and was scored as 2. A moribund state was scored as 1; death was scored as 0. The investigator was blinded to the group allocation during the experiment and when assessing the outcome.

### Gut Microbiota Analysis

Female Balb/c mice aged 6–8 weeks old were used in the experiments. Twenty-four mice were randomly divided into four groups (EF, EF+P, P, and WT) that contained six animals each. The mice in the EF group were challenged i.p. with 2 × 10^9^ CFU of *E. faecalis* strain 002 per mouse. The mice in the EF+P group were challenged with 002 as in the EF group and then injected i.p. with 4 × 10^5^ PFU of EF-P29 after 1 h. The mice in the P group were injected i.p. with 2 × 10^9^ PFU EF-P29, and the mice in the WT group were not treated. The feces of each mouse was collected at 24 h post-infection. The feces samples (24 in all) were snap-frozen in liquid nitrogen and then stored at -80°C until DNA extraction.

For each fecal sample, total genomic DNA was extracted using a QIAamp DNA stool mini kit (Qiagen, West Sussex, United Kingdom) according to the manufacturer’s instructions. DNA was frozen at -80°C prior to PCR amplification. Broadly conserved primers with the barcodes 515F (5′-GTGCCAGCMGCCGCGGTAA-3′) and 806R (5′-GGACTACHVGGGTWTCTAAT-3′) (Evans et al., 2014) were used to amplify the V4 region (300 bp) of 16S rRNA. All PCRs were performed using Phusion@ High-Fidelity PCR Master Mix (New England Biolabs). The PCR products were purified using a Qiagen Gel Extraction Kit (Qiagen, Germany). Sequencing libraries were generated using a TruSeq^®^ DNA PCR-Free Sample Preparation Kit (Illumina, USA) according to the manufacturer’s recommendations, and index codes were then added. Library quality was assessed using a Qubit@ 2.0 Fluorometer (Thermo Scientific) and an Agilent Bioanalyzer 2100 system. Finally, the library was sequenced on an Illumina HiSeq2500 platform to generate 250 bp paired-end reads.

Paired-end reads were assigned to the samples based on their unique barcode and then truncated by cutting off the barcode and primer sequence. The paired-end reads were then merged using FLASH (V1.2.7^2^) (Magoc and Salzberg, 2011). Quality filtering of the raw tags was performed under specific filtering conditions to obtain high-quality clean tags (Bokulich et al., 2013) using the QIIME (V1.7.0^3^) (Caporaso et al., 2010). The tags were compared to the reference database (Gold database^4^) using the UCHIME algorithm (UCHIME Algorithm^5^) (Edgar et al., 2011) to detect chimeric sequences, and the chimeric sequences were then removed (Haas et al., 2011).

Sequences analyses were performed using Uparse software (Uparse v7.0.1001^6^) (Edgar, 2013). Sequences with ≥97% similarity were assigned to the same OTUs. The representative sequence for each OTU was screened for further annotation. Multiple sequence alignments were constructed using MUSCLE software (Version 3.8.31^7^) (Edgar, 2004). Alpha diversity was applied to analyze the complexity of species diversity in each sample using six indices, including Observed-species, Chao1, Shannon, Simpson, ACE, and Good-coverage. All of the indices for our samples were calculated using QIIME (Version 1.7.0) and displayed using R software (Version 2.15.3). A beta diversity analysis was used to evaluate differences in species complexity between the samples. This consisted of principal component analysis (PCA), principal coordinate analysis (PCoA), and non-metric multidimensional scaling (NMDS), which were calculated using QIIME software (Version 1.7.0).

### Data Analysis

SPSS version 13.0 (SPSS, Inc., Chicago, IL, USA) was used to statistically analyze the experimental data using one-way analysis of variance. A *P*-value < 0.05 was considered to indicate statistical significance. The error bars represent the standard deviation of the mean.

## Results

### Identification and Antibiotic Resistance of *E. faecalis* Strains

The 16S rRNA and SodA gene fragments of 40 putative *E. faecalis* strains were amplified using PCR. Their sizes were approximately 733 and 360 bp according to gel electrophoresis and sequencing, respectively. The BLAST analysis showed that the SodAs of these 40 strains were more than 99% identical to those of *E. faecalis* strains that were identified in the GenBank database (such as CP008816.1 and AE016830.1).

Most of the *E. faecalis* isolates were sensitive to ampicillin and fosfomycin and resistant to erythromycin, streptomycin, chloramphenicol, tetracycline, ciprofloxacin, rifampicin, daptomycin, and linezolid (Supplementary Table S1). Interestingly, 22 of the strains were vancomycin-resistant. The results of RAPD indicated that there was genetic diversity among the *E. faecalis* isolates (Supplementary Figure S1). However, several of the isolates also had highly similar fingerprints (e.g., 2GF-2 and 2GF-25, GF-2 and GF-27, and GF26 and E028), and there was limited correspondence between genetic type and the observed pattern of antibiotic susceptibility.

### Isolation and Characterizations of EF-P29

**FIGURE 1:**
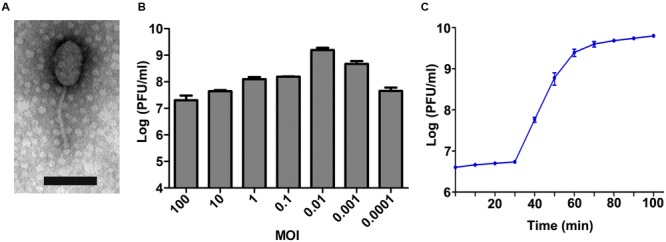
**Characteristics of phage EF-P29. (A)** The morphology of phage EF-P29. An EF-P29 phage was negatively stained with 2% phosphotungstic acid (PTA) and examined using transmission electron microscopy (TEM) at an accelerating voltage of 80 kV. The scale bars represent 100 nm. **(B)** Titers of EF-P29 under different multiplicities of infection (MOI). At a MOI of 0.01, the EF-P29 titers reached >10^9^ PFU/ml. **(C)** One-step growth curve for EF-P29 in *Enterococcus faecalis* GF29. EF-P29 was added at a MOI of 0.01. The values indicate the means and standard deviations (SD) (*n* = 3).

In BHI agar plates that were covered with a bacterial lawn of *E. faecalis* GF29, clear plaques were observed wherever the phage lysate was spotted. This verified that the lysate did contain phages. After purification, the host ranges of five phages were determined, and the phage with the broadest host spectrum was chosen and named EF-P29. As shown in Supplementary Table S2, the spot tests showed that EF-P29 lysed 17 (42.5%) of the 40 *E. faecalis* isolates that were tested, and efficiency of plating tests showed that it produced large, clear plaques on 11 of the *E. faecalis* strains (27.5%). Based on the limited data, the phage EF-P29 was unable to lyse *E. faecium* or any other tested species (Supplementary Table S2). The results of electron microscopy showed that phage EF-P29 had an icosahedral head and a non-contractile tail, suggesting that it is a member of the *Siphoviridae* family, as shown in **Figure 1A**. The width of the head was approximately 60 ± 10 nm, its length was approximately 90 ± 10 nm, and its tail was approximately 105 ± 5 nm long. When the MOI was 0.01, the phage titer was the highest, reaching >10^9^ PFU/ml (**Figure 1B**). A one-step growth curve experiment (MOI = 0.01) revealed that the latent periods was approximately 30 min long and the burst size was estimated at approximately 90 PFU per infected cell, as shown in **Figure 1C**.

### General Genome Description

The complete genome sequence of EF-P29 is available at GenBank under the accession number GKY303907. The EF-P29 phage genome is a 58,984 bp contiguous sequence of linear, double-stranded DNA with an overall G+C content of 39.97%. The complete genome of EF-P29 encodes 101 putative ORFs, but no tRNA was identified. The arrangement of putative ORFs at the whole-genome level was mapped (Supplementary Figure S2A). The entire genome can be approximately divided into two distinct regions that are divergently transcribed, as shown in Supplementary Figure S2A and Table S3. As shown in Supplementary Table S3, of the identified ORFs, 39 gene products that presented an obvious similarity to proteins of known function were tentatively assigned a corresponding function. In addition, two gene products were unique and produced no database match to any publicly available viral or prokaryotic sequence. According to the functions identified for all of the predicted ORFs, the genome of EF-P29 comprises three discrete modules, including nucleotide metabolism and replication, lysis, and morphogenesis, but does not possess a lysogeny module. Of these modules, the lysis module is the smallest one, and it includes only two discrete ORFs (ORFs 62 and 81).

**FIGURE 2:**
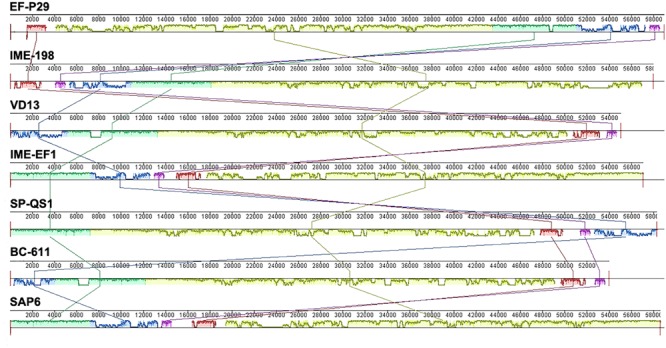
**A comparison of the genomic locations of homologous open reading frames (ORFs).** Nucleotide base pairs are indicated between gray lines for each phage genome. Pairs of homologous ORFs between two phages were identified using BLASTN analysis.

Phage EF-P29 shared identity at the genome level with several other *E. faecalis* phages, such as IME198, VD13, IME-EF1, BC-611, SAP6 and the *Streptococcus* phage SP-QS1, as shown in Supplementary Table S4 and **Figure 2**. Additionally, several ORFs showed homology at the amino acid level with putative functional proteins in other phages that infect other species, such as *Bacillus* and *Listeria*.

Open reading frame 79 and ORF 80 of EF-P29 shared high identity at both the gene sequence and amino acid level with two components of the terminase large subunit (originating from the *E. faecalis* phages IME-EF1, IME198, and SAP6 and the *Streptococcus* phage SP-QS1). However, there is a 401 bp insertion sequence between ORF 79 and ORF 80 of EF-P29 that is not present in these homologous and whole terminase large subunit genes (Supplementary Figure S2B).

### The *In Vitro* Bactericidal Effect of the Phage EF-P29

**FIGURE 3:**
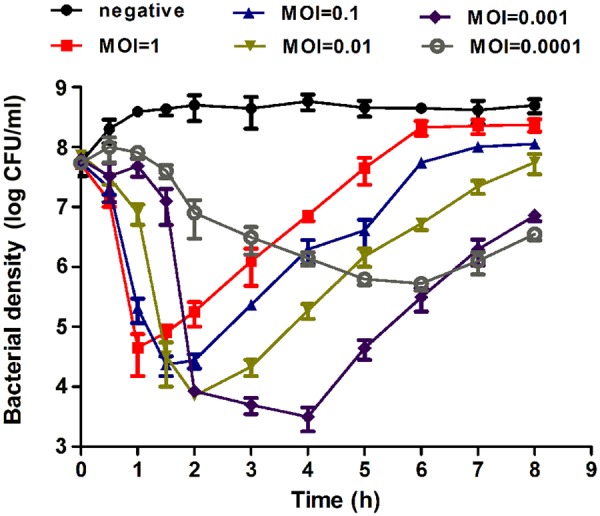
**Bactericidal activity of EF-P29 at different multiplicities of infection *in vitro*.** The VREF 002 strain was lysed by phage EF-P29 in BHI medium at 37°C. CFU counts of the uninfected control culture and the parallel cultures that were infected with the phage at different MOI were measured over time. No phage was added in the negative control. The values represent the means and SD (*n* = 3).

The effect of the phage EF-P29 on the growth of VREF strain 002 was tested *in vitro*. As shown in **Figure 3**, the bacteria numbers increased continuously in the control group. In contrast, the VREF strain 002 showed a rapid decrease after incubation with EF-P29 at a MOI of 1, 0.1, 0.01, and 0.001. EF-P29 at the higher MOI showed more efficiently bactericidal effect. The bacteria numbers of the phage-treated cultures reached the lowest peak at different time (MOI = 1, reached 4.6 log units at 1 h; MOI = 0.1, reached 4.3 log units at 1.5 h; MOI = 0.01, reached 3.9 log units at 2 h; MOI = 0.001, reached 3.5 log units at 4 h). From this peak point, the bacteria numbers began to increase, suggesting the phage-resistant bacteria proliferated massively. In contrast, when EF-P29 was added at the MOI of 0.0001, the bacteria numbers decreased slowly (reached the lowest peak of 5.7 log units at 6 h).

### Elimination of VREF by EF-P29 in a Mouse Model of Bacteremia

**FIGURE 4:**
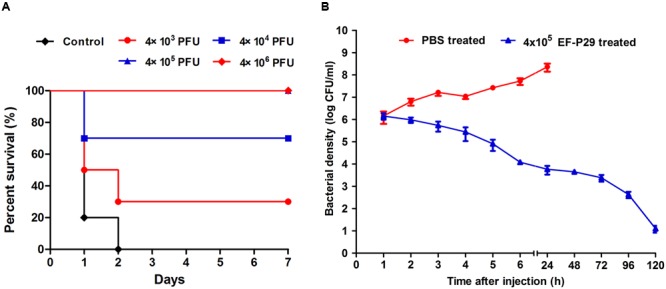
**EF-P29 rescued mice from a lethal VREF 002 infection. (A)** Survival rates. Mice were inoculated intraperitoneally with 2 × 10^9^ CFU of the VREF 002 strain. One hour later, different phage doses were introduced intraperitoneally to treat the challenged mice. The control mice were treated with PBS under the same conditions. **(B)** Colony counts in peripheral blood samples. EF-P29 (4 × 10^5^ PFU) or PBS was administered intraperitoneally at 1 h after challenge with 2 × 10^9^ CFU of the VREF 002 strain. At the indicated times, the bacterial counts (CFU/ml) in the mice were determined from peripheral blood samples (10 μl) that were obtained from the caudal vein. The means and SD are shown as points with error bars (*n* = 5).

**FIGURE 5:**
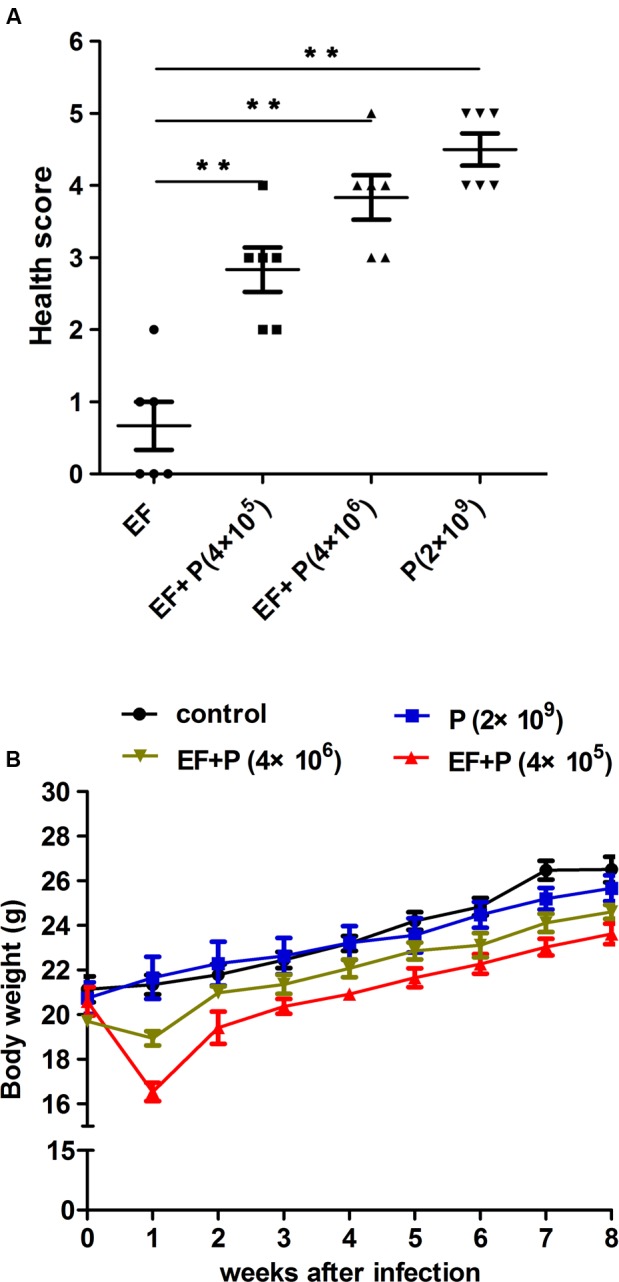
**Comparison of mouse mice states. (A)** The health score. The mice were scored for their state of health on a scale of 5 to 0 based on disease progression at 24 h after treatment. A score of 5 indicates normal health and an unremarkable condition. Slight illness was defined as decreased physical activity and ruffled fur and was scored as 4. Moderate illness was defined as lethargy and a hunched back and was scored as 3. Severe illness was defined as the aforementioned signs plus exudative accumulation around partially closed eyes and was scored as 2. A moribund state was scored as 1; death was scored as 0. Each dot indicates the health score of a single mouse. EF, ●; EF+P (4×10^5^), ■; EF+P (4×10^6^), ▲; P (2×10^9^), ▼. ^∗∗^*P* < 0.01. **(B)** Body weight gain. Body weight was recorded weekly.

Injecting ≥1 × 10^9^ CFU/mouse of VREF 002 (i.p.) was sufficient to produce bacteremia and caused a 100% mortality rate within 2 days (as shown in **Figure 4A**). The average MLD of 002 was approximately 1 × 10^9^ cells. When 2×MLD was used as the challenge dose, high bacteria counts (>10^6^ CFU/ml) were observed in blood samples (as shown in **Figure 4B**), and the infection became systemic within 1 h after challenge. Three of the mice treated with this dose had died and the others were dying within 24 h after challenge, as shown in **Figure 5A**.

Consequently, different doses of EF-P29 were administered (i.p.) at 1 h after the challenge inoculation. A dose-dependent effect was observed in the state of the animals after 24 h. All of the mice recovered and bacteremia was substantially reduced when 4 × 10^5^ and 4 × 10^6^ PFU of EF-P29 were administered i.p. at 1 h after VREF challenge (**Figures 4A,B**). When the bacteremic mice were treated with 4 × 10^5^ PFU/mouse, their health scores were higher than those of the control group, and their body weights recovered after phage treatment (**Figures 5A,B**). When the dose of phage was increased to 4 × 10^6^ PFU/mouse, the mice appeared healthier. A lower dose of phage (4 × 10^4^ PFU/mouse) was sufficient to protect seven out of 10 tested mice, while a dose of 4 × 10^3^ PFU protected only three out of 10 tested mice (**Figure 4A**). The mice that were treated with EF-P29 (4 × 10^5^ PFU/mouse) at 1 h after inoculation exhibited a drop in blood bacterial counts that reached approximately 3.8 log units at 24 h after treatment with EF-P29 (**Figure 4B**). In contrast, the bacterial loads in the control bacteremic mice, which were treated with PBS instead of phages, had reached approximately 8.5 log units at 24 h after treatment.

### The Influence of Phage Therapy on the Mouse Gut Microbiota

**FIGURE 6:**
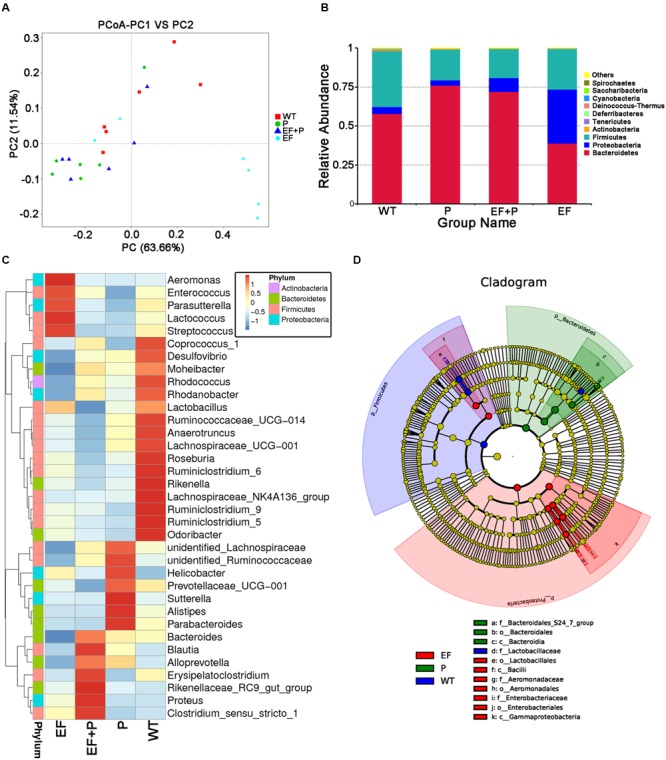
**The effects of *E. faecalis* challenge and phage therapy on the composition of the gut microbiota.** The composition of the gut microbiota in the feces of challenged and normal mice that were treated with or without phage EF-P29 was analyzed using 16S rRNA gene sequencing (*n* = 6 for each group). **(A)** A plot was generated using the weighted version of the UniFrac-based PCoA. **(B)** Bacterial taxonomic profiling at the phylum level of intestinal bacteria identified in different mouse groups. **(C)** Heatmapping shows that the abundance of intestinal bacteria at the genus level was significantly altered by treatment with phage EF-P29 in challenged and normal mice. **(D)** The cladogram demonstrates the phylogenetic distribution of microbial lineages in fecal samples obtained from differently treated mice.

The intestinal microflora in the VREF challenge and phage-treated groups were analyzed. Because the experimental mice in the EF group (challenge but no treatment) would have all died at later timepoints, fecal samples were obtained from the mice at 24 h after treatment. A total of 1,613,040 previously selected effective tags were generated, and each fecal sample (*n* = 6 in each group) produced an average of 53,768 effective tags. The average OTUs of each sample was approximately 550. The rarefaction curve and rank abundance indicated that the sequencing depth covered rare new phylotypes and most of the observed diversity (Supplementary Figures S3A,B). UniFrac-based PCoA revealed that there was a distinctly clustered microbiota composition in each treatment group (**Figure 6A**). When the mice were challenged with VREF 002, the microbial composition of 4/6 of the mice shifted significantly away from the composition observed in the WT group. However, no significant shift in microbial composition was observed across the clusters observed in the P, EF+P, and WT groups, which could not be completely separated.

**FIGURE 7:**
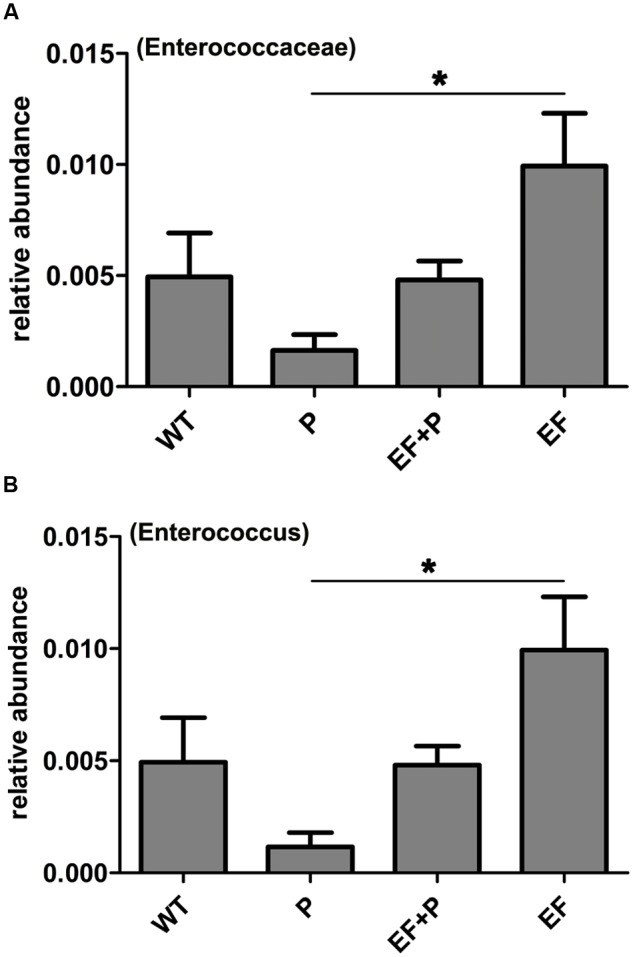
**The effects of *E. faecalis* challenge and phage therapy on members of the genus *Enterococcus* within the family *Enterococcaceae* in the gut microbiota. (A)** The relative abundance of members of the family *Enterococcaceae* in the different mouse groups. **(B)** The relative abundance of the genus *Enterococcus* in the different mouse groups. The values represent the means and SD (*n* = 6). ^∗^*P* < 0.05.

Taxonomic profiling demonstrated that when VREF 002 was injected i.p. into the mice, there were initially more Firmicutes and Bacteroidetes and fewer Proteobacteria increased than were observed in the normal group (**Figure 6B**). Notably, VREF 002 challenge altered the normal balance of the gut microbiota. Specifically, bacteria in the genus *Enterococcus* were present in higher numbers in the challenge group than in the normal mice (**Figures 7A,B**). Moreover, in comparison to normal mice, the VREF challenge mice also exhibited an enhanced variety of bacterial genera, including *Streptococcus, Aeromonas, Parasutterella*, and *Lactococcus* (**Figures 6C,D** and Supplementary Figure S4). At the same time, *Coprococcus*_1, *Desulfovibrio, Moheibacter, Rhodococcus, Rhodanobacter, Lactobacillus, Ruminococcae, Anaerotruncus, Lachnospiraceae, Roseburia, Ruminiococcaceae*_UCG-014, *Rikenella, Lachnospiraceae*_UCG-001, *Ruminiclostridium*_9, *Ruminiclostridium*_5, and *Odoribacter* genera were significantly reduced by VREF 002 challenge (**Figure 6C**). However, in the phage-treated group (4 × 10^5^ PFU/mouse), balance was largely restored in the gut microbiota. The changes that had been observed in Bacteroidetes and Proteobacteria were reversed (**Figure 6B**), and phage treatment reduced the level of the genus *Enterococcus* and family *Enterococcaceae* that were observed in the challenge mice to a level similar to that in the normal mice (**Figures 6C, 7A,B** and Supplementary Figure S5). These results suggest that the observed increases in *Enterococcus* in the gut microbiota were reduced by treatment with 4 × 10^5^ PFU/mouse EF-P29. Additionally, all of the genera that were different in the challenge group compared to the normal group, including *Streptococcus, Aeromonas, Parasutterella, Lactococcus, Coprococcus*_1, *Desulfovibrio, Moheibacter, Rhodococcus, Rhodanobacter, Lactobacillus, Ruminococcae, Anaerotruncus, Lachnospiraceae, Roseburia, Ruminiococcaceae*_UCG-014, *Rikenella, Lachnospiraceae*_UCG-001, *Ruminiclostridium*_9, *Ruminiclostridium*_5, and *Odoribacter*, returned to normal levels after treatment with 4 × 10^5^ PFU/mouse EF-P29 (**Figure 6C**). Collectively, these results show that the phage EF-P29 modulates the gut microbiota in VREF-challenged mice by suppressing the genus *Enterococcus*, which results in a microbiota composition that is similar to that observed in normal mice.

When a large dose of phage EF-P29 (4 × 10^9^ PFU/mouse) was administered to normal mice, bacteria in the genus *Enterococcus* were significantly reduced in number, which substantially altered the composition of the gut microbiota from that observed in the normal group (**Figures 6C, 7A,B**). Nevertheless, there was no significant difference in health scores or body weight between the normal mice that were treated with the large dose of EF-P29 (4 × 10^9^ PFU/mouse) and normal untreated mice, as shown in **Figures 5A,B**.

## Discussion

Because there has been a rise in the prevalence of multidrug-resistant strains of *E. faecalis*, novel therapeutic approaches to combat this pathogen are needed. Phages have long shown promise as potential antibacterial therapeutics, and there has therefore been a revival in phage therapy over the last several years (Duerkop et al., 2016). In the present study, we describe the isolation of a new *E. faecalis* phage that we have named EF-P29. Its small optional MOI, short latency period and fast release period indicate that phage EF-P29 possesses a highly efficient ability to infect *E. faecalis*. EF-P29 can lyse not only antibiotic-sensitive *E. faecalis* strains but also antibiotic-resistant *E. faecalis* strains, including vancomycin-resistant strains. Our genome sequencing analysis did not reveal any putative virulence factors or antibiotic resistance genes. All these characteristics indicates that EF-P29 meets additional major prerequisites for use as a candidate therapeutic phage, and it should therefore be considered as a candidate phage and further studied to determine its therapeutic applications (Uchiyama et al., 2008; Niu et al., 2012; Gu et al., 2016).

EF-P29 shared high similarity with several other *E. faecalis* phages, such as IME198, VD13, IME-EF1, BC-611, and SAP6. Interestingly, these *E. faecali*s phages share high similarity with *Streptococcus* SP-QS1 (GenBank: HE962497). This similarity suggests that these phages are closely related (Gu et al., 2013). Indeed, *E. faecalis* was formerly classified as a member of the Group D *Streptococcus* system (Zhang et al., 2013).

A BLAST analysis showed that ORF 79 and ORF 80 of EF-P29 most likely encode two components of the terminase large subunit. We identified a small insertion between the EF-P29 ORF 79 and ORF 80 genes. There are also 1017 and 990 bp insertion sequences in the terminase large subunit genes in the phages VD13 and BC-611, respectively. However, the 401 bp insertion fragment identified in EF-P29 did not contain a complete ORF. One ORF encodes a homing endonuclease (approximately 200 amino acids) in each insertion sequence in each of the VD13 and BC-611 terminase large subunit genes. The large terminase subunit gene in the staphylococcal phage Twort also contains three introns that encode homing endonuclease-like genes in the HNH superfamily (Kwan et al., 2005; Gu et al., 2013). Although there is no homing endonuclease gene between ORF 79 and ORF 80, there are six ORFs in the other sites of the phage EF-P29 genome, including the ORFs 42, 55, 59, 93, 98, and 99, and these share identity with the HNH homing endonucleases in other phages. These putative proteins may be responsible for intron mobility.

When the phage EF-P29 was incubated with a VREF strain at a higher MOI (MOI = 1), phage-resistance mutations occurred quickly in *E. faecalis*. The high titer of the phage may have exert strong selective pressure on *E. faecalis* to develop phage resistance (Duerkop et al., 2016). Lower MOI allows wild-type (phage-sensitive) bacteria to persist for a longer period of time, which then outcompete (or at least suppress) the growth of phage-resistant bacteria. The rapid development of phage resistance is one of several barriers to therapeutically deploying phages (Alexandra, 2013). However, several earlier studies have reported that the appearance of phage-resistant mutants was not considered an important consideration because resistant variants tended to be avirulent and were easily removed by phagocytes and the immune system (Smith and Huggins, 1983; Tanji et al., 2005; Zahid et al., 2008). Previous studies have indicated that the phage cocktail can delay the appearance of phage-resistant variants and enhance treatment efficacy (Tanji et al., 2004; Gu et al., 2012). Thus, we will isolate and screen additional *E. faecalis* phages to test the effectiveness of phage cocktails in future studies.

Several reports have described successful *E. faecalis* phage therapies. Administering the bacteriophage IME-EF1 (10^10^ PFU) at 30 min post-inoculation resulted in a therapeutic efficacy and a survival rate of only 60% (Zhang et al., 2013). A single i.p. injection of 3 × 10^8^ PFU of the phage ENB6 at 45 min after bacterial challenge was sufficient to rescue 100% of the animals (Biswas et al., 2002), and the phage ΦEF24C (>10^8^ PFU) rescued 100% of septic BALB/c mice (Uchiyama et al., 2008). In comparison, a single small dose of EF-P29 (4 × 10^5^ PFU) was sufficient to protect all of the inoculated mice against VREF-induced bacteremia.

There are many common commensal bacteria in the intestinal tract. Any factor that is capable of destroying these commensal bacteria can affect the balance of the gut microbiota. A recent study showed that administering antibiotics significantly impacted host physiology by reducing gut microbiota diversity, bile acid metabolism, and insulin sensitivity (Vrieze et al., 2014). Another article demonstrated that antibiotic-induced imbalances in the gut microbiota aggravated the accumulation of cholesterol and liver injuries in rats that were fed a high-cholesterol diet (Hu et al., 2015). *E. faecalis* is a common commensal bacterium that originates from humans and animals that commonly and primarily inhabits the lower intestinal tract. Thus, whether phages that specifically target *E. faecalis* can evacuate the *E. faecalis* that inhabit the intestines to thereby affect the balance of the gut microbiota should be evaluated. To our knowledge, only Duerkop et al. (2016) has reported that phage ΦVPE25 can modestly reduce the number of *E. faecalis* in the intestines of gnotobiotic mice at 24 h after oral administration. Thus, in the present study, we determined the influence of VREF challenge and phage treatment on the gut microbiota.

The flora ratio in the feces may reflect the composition of the gut microbiota in mice (Chang et al., 2015; Sonnenburg et al., 2016). Thus, to understand the effect of *E. faecalis* challenge and treatment with phage EF-P29 on the gut microbiota, we analyzed fecal samples. When the mice were challenged with a 2×MLD VREF strain, the number of bacteria in the genus *Enterococcus* in the feces increased significantly and all of these mice eventually die. *E. faecalis* is widely accepted to be the principle member of the genus *Enterococcus*. These data therefore indicate that the challenged *E. faecalis* strain was capable of entering and colonizing the gut. *E. faecalis* is an opportunistic pathogen, and most *E. faecalis* infection-related disease is the result of *E. faecalis* entering sites where it does not belong (Prematunge et al., 2016). This occurs because there is no significant boundary between normal *E. faecalis* and pathogenic *E. faecalis*.

We found that EF-P29 not only cleared i.p. administered *E. faecalis* from the blood but also most likely eliminated the *E. faecalis* in the gut. Additionally, a high dose of phage (10^9^ PFU) had an inhibitory effect on the genus *Enterococcus* in the feces. Because EF-P29 has a broad host range *in vitro*, it may also infect normal *E. faecalis* strains in gut. Thus, the prophylactic administration of phages should not be encouraged, especially phages that can infect a number of normal flora, such as *E. faecalis* phages, as has previously been reported (Duerkop et al., 2016). We also found that the variety of species in the genus *Enterococcus* can induce changes in many bacteria in other families that reside in the gut. We hypothesize that when *E. faecalis* is altered, the balance of gut microbiota communities is disturbed. When *E. faecalis* returned to a normal level following treatment with the phage EF-P29, most of the families that experienced changes in the gut were substantially recovered. Although a large dose of EF-P29 did affect the balance of the gut microbiota community, body weight and health levels were not significantly influenced.

To our knowledge, most of reported *E. faecalis* phages can only lyse *E. faecalis* strains. However, *E. faecalis* phages IME-EF1 (Zhang et al., 2013), φ4D (Parasion et al., 2012), and EFDG1 (Otawa et al., 2012) have been reported to be able to lyse both of *E. faecalis* and *E. faecium*. A broad bactericidal spectrum against the target pathogenic bacterium is considered an essential prerequisite of therapeutic candidates (Fischetti, 2010; Young, 2013). However, both of *E. faecium* and *E. faecalis* are usually considered harmless commensal bacteria in healthy humans and animals. *E. faecalis* phages with multi-species bactericidal activity may also evacuate normal *E. faecium* in the patient when eliminating pathogenic *E. faecalis*, which is likely cause bigger impact on the gut microbiota.

All of these data suggest phage therapies that are aimed at treating opportunistic pathogens, such as *E. faecalis*, are feasible, as long as the dose of phage is controlled to attenuate the influence on the gut microbiota.

## Ethics Statement

All experimental animal procedures were performed in strict accordance with the Regulations for the Administration of Affairs Concerning Experimental Animals that was approved by the State Council of People’s Republic of China (1988.11.1) and with approval of the Animal Welfare and Research Ethics Committee of Jilin University.

## Author Contributions

MC, JMG and WH drafted the main manuscript and performed the data analysis. MC, JL, YZ, LH, PG, RC, LZ, HZ, JLG, YJ, ZG, XF, CS, YY, and LL planned and performed experiments. WH and JMG were responsible for experimental design; and WH and JMG were responsible for guiding and supporting the experiments and manuscript revisions.

## Conflict of Interest Statement

The authors declare that the research was conducted in the absence of any commercial or financial relationships that could be construed as a potential conflict of interest.

## Footnotes

1 http://wishart.biology.ualberta.ca/cgview/

2 http://ccb.jhu.edu/software/FLASH/

3 http://qiime.org/index.html

4 http://drive5.com/uchime/uchime_download.html

5 http://www.drive5.com/usearch/manual/uchime_algo.html

6 http://drive5.com/uparse/

7 http://www.drive5.com/muscle/
