# Supplementary material for: The Bacteriophage EF-P29 Efficiently Protects against Lethal Vancomycin-Resistant Enterococcus faecalis and Alleviates Gut Microbiota Imbalance in a Murine Bacteremia Model
Source: Front Microbiol. 2017 May 9;8:837. doi: 10.3389/fmicb.2017.00837 (PMC5423268; doi:10.3389/fmicb.2017.00837)
Supplement: Supplementary file 1 [file Table_1.DOC]

**Table S1. The antibiotic-resistance of *E. faecalis* isolates.**

|  | Vancomycin | Erythromycin | Streptomycin | Chloramphenicol | Ampicillin | Gentamicin | Tetracycline | Nitrofurantoin | Ciprofloxacin | **Rifampicin** | Fosfomycin | Daptomycin | Linezolid |
| --- | --- | --- | --- | --- | --- | --- | --- | --- | --- | --- | --- | --- | --- |
| ATCC51299 | R | R | R | R | S | R | R | S | I | R | S | R | S |
| ATCC29212 | S | S | R | S | S | S | S | R | S | S | S | R | I |
| M1 | I | R | R | R | S | S | R | R | R | S | S | R | R |
| M2 | I | R | R | S | R | S | R | S | R | R | R | R | I |
| M4 | I | R | R | I | S | S | R | S | I | R | S | R | R |
| N9 | I | R | R | R | S | R | R | S | R | I | I | R | R |
| N10 | R | R | R | R | S | R | R | S | R | R | I | R | I |
| GF2 | I | R | R | R | S | R | R | I | R | R | I | R | R |
| GF12 | R | R | R | R | S | R | R | S | R | R | S | R | S |
| GF23 | I | R | R | R | S | R | R | S | R | R | S | R | S |
| GF25 | R | R | R | I | S | S | R | I | I | R | S | R | S |
| GF26 | I | R | R | S | R | R | R | R | R | R | S | R | I |
| GF27 | I | R | R | R | S | S | R | I | R | R | S | R | R |
| GF29 | R | R | R | R | S | R | R | S | R | R | S | R | R |
| 2GF-7 | R | R | R | S | S | R | R | S | I | R | S | R | I |
| 2GF-25 | R | R | R | S | S | R | R | S | I | R | S | R | I |
| E014 | R | R | R | R | S | R | R | S | R | R | S | R | R |
| E028 | R | R | R | S | S | R | R | I | I | R | S | R | R |
| Z2 | R | R | R | S | S | R | R | R | R | I | S | R | R |
| ZC28 | R | R | R | R | S | S | R | I | R | R | S | R | R |
| ZI4 | R | R | R | R | S | R | R | R | R | R | I | R | R |
| ZJ21 | S | R | R | R | S | R | R | S | R | R | I | R | R |
| ZJ28 | I | R | R | R | S | R | R | S | R | I | S | R | I |
| 2NH4 | R | R | R | S | S | R | S | R | R | R | S | R | S |
| 5-1 | S | R | R | S | S | R | R | S | I | R | S | R | R |
| 6-1 | S | R | R | S | S | R | R | R | R | R | S | R | S |
| 002 | R | I | R | I | S | S | R | S | R | R | S | R | S |
| 281 | R | R | R | I | S | R | R | S | R | R | S | R | I |
| 333 | R | I | R | S | S | S | R | S | R | R | S | R | R |
| 410 | R | R | R | S | R | R | I | R | R | R | S | R | R |
| 436 | R | R | R | S | R | R | R | I | R | R | S | R | R |
| 815 | R | R | R | S | S | S | I | S | R | R | S | R | R |
| 1545 | R | I | R | I | S | S | R | S | I | R | S | R | I |
| 1547 | R | R | R | R | S | R | S | S | R | I | S | R | R |
| 1549 | R | R | R | I | S | S | R | S | I | R | S | R | R |
| 1556  FA1  FA2  FA3  FA4 | S  S  S  S  S | R  R  S  R  R | R  R  R  R  R | S  I  S  R  I | R  S  S  R  R | S  S  S  S  S | R  R  S  R  R | S  S  S  R  R | R  S  S  R  R | R  R  I  R  R | S  S  S  I  I | S  R  R  I  R | I  S  S  S  S |

S, Susceptible; I, Intermediate; R, Resistance.
