# Supplementary material for: The Bacteriophage EF-P29 Efficiently Protects against Lethal Vancomycin-Resistant Enterococcus faecalis and Alleviates Gut Microbiota Imbalance in a Murine Bacteremia Model
Source: Front Microbiol. 2017 May 9;8:837. doi: 10.3389/fmicb.2017.00837 (PMC5423268; doi:10.3389/fmicb.2017.00837)
Supplement: Supplementary file 2 [file Table_2.DOCX]

**Table S2. Lytic spectrum of EF-P29.**

| Bacterial strains | Spot^6^ | Forms plaques^6^ | Bacterial strains | Spot^6^ | Forms plaques^6^ |
| --- | --- | --- | --- | --- | --- |
| *Enterococcus faecalis* M1^1^ | N | N | *Enterococcus faecium* T1^1^ | N | N |
| *E. faecalis* M2^1^ | N | N | *E. faecium* E030^1^ | N | N |
| *E. faecalis* M4^1^ | N | N | *E. faecium* E032^1^ | N | N |
| *E. faecalis* N9^1^ | N | N | *E. faecium* E016^1^ | N | N |
| *E. faecalis* N10^1^ | Y | Y | *E. faecium* 4P-SA^1^ | N | N |
| *E. faecalis* GF-2^1^ | Y | Y | *E. faecium* FC11^1^ | N | N |
| *E. faecalis* GF-12^1^ | Y | Y | *E. faecium* J1^1^ | N | N |
| *E. faecalis* GF-23^1^ | Y | Y | *E. faecium* E042^1^ | N | N |
| *E. faecalis* GF-25^1^ | Y | N | *E. faecium* E044^1^ | N | N |
| *E. faecalis* GF-26^1^ | N | N | *E. faecium* FC18^1^ | N | N |
| *E. faecalis* GF-27^1^ | N | N | *E. faecium* E038^1^ | N | N |
| *E. faecalis* GF-29^1^ | Y | Y | *E. faecium* FC9^1^ | N | N |
| *E. faecalis* 2GF-2^1^ | Y | N | *E. faecium* 2NH23^1^ | N | N |
| *E. faecalis* 2GF-25^1^ | Y | N | *E. faecium* E031^1^ | N | N |
| *E. faecalis* E014^1^ | N | N | *E. faecium* 1543^2^ | N | N |
| *E. faecalis* E028^1^ | Y | N | *E. faecium* 081^2^ | N | N |
| *E. faecalis* Z2^1^ | N | N | *E. faecium* 378^2^ | N | N |
| *E. faecalis* ZC28^1^ | N | N | *E. faecium* 383^2^ | N | N |
| *E. faecalis* ZJ4^1^ | Y | Y | *E. faecium* 363^2^ | N | N |
| *E. faecalis* ZJ21^1^ | N | N | *E. faecium* 327^2^ | N | N |
| *E. faecalis* ZJ28^1^ | Y | N | *E. faecium* 483^2^ | N | N |
| *E. faecalis* 2NH-4^1^ | N | N | *E. faecium* 353^2^ | N | N |
| *E. faecalis* 5-1^1^ | N | N | *E. faecium* 106^2^ | N | N |
| *E. faecalis* 6-1^1^ | N | N | *Pseudomonas aeruginosa* ATCC27853^3^ | N | N |
| *E. faecalis* ATCC51299^3^ | N | N | *P. aeruginosa* 5^5^ | N | N |
| *E. faecalis* ATCC29219^3^ | Y | Y | *P. aeruginosa* 8^5^ | N | N |
| *E. faecalis* 002^2^ | Y | Y | *Escherichia coli* ATCC 25922^3^ | N | N |
| *E. faecalis* 281^2^ | N | N | *E.coli* O157^5^ | N | N |
| *E. faecalis* 333^2^ | Y | Y | *E.coli* 01^1^ | N | N |
| *E. faecalis* 410^2^ | N | N | *Staphylococcus aureus* ATCC 25923^3^ | N | N |
| *E. faecalis* 436^2^ | Y | N | *S.aureus* USA300 -TCH1516^3^ | N | N |
| *E. faecalis* 815^2^ | N | N | *S.aureus* YB57^5^ | N | N |
| *E. faecalis* 1545^2^ | Y | Y | *Bacillus subtilis* ATCC14579^3^ | N | N |
| *E. faecalis* 1547^2^ | N | N | *B.subtilis* KU2^5^ | N | N |
| *E. faecalis* 1549^2^ | Y | Y | *B.subtilis* 07^5^ | N | N |
| *E. faecalis* 1556^2^ | N | N | *Streptococcus sp.* CVCC606^4^ | N | N |
| *E. faecalis* FA1^1^ | Y | N | *Streptococcus sp.*167^5^ | N | N |
| *E. faecalis* FA2^1^ | N | N | *Streptococcus sp.*168^5^ | N | N |
| *E. faecalis* FA3^1^ | N | N | *Klebsiella pneumoniae* BAA-2146^3^ | N | N |
| *E. faecalis* FA4^1^ | N | N | *K. pneumoniae* 16^5^ | N | N |
|  |  |  | *K. pneumoniae* 18^5^ | N | N |

^1^ isolated from patients at the First Hospital of Jilin University (Changchun, Jilin province, China); ^2^ given by professer Yigang Tong; ^3^ purchased from American Type Culture Collection (ATCC); ^4^ purchased from China Institute of Veterinary Drug Control (CVCC); ^5^ Collected in laboratory ; ^6^ Y, yes; N, no.
