# Supplementary material for: The Bacteriophage EF-P29 Efficiently Protects against Lethal Vancomycin-Resistant Enterococcus faecalis and Alleviates Gut Microbiota Imbalance in a Murine Bacteremia Model
Source: Front Microbiol. 2017 May 9;8:837. doi: 10.3389/fmicb.2017.00837 (PMC5423268; doi:10.3389/fmicb.2017.00837)
Supplement: Supplementary file 3 [file Table_3.DOCX]

**Table S3. General features of the putative ORFs from phage EF-P29 with the best matches in the database.**

| ORF* | Start codon | Bp | | Protein size (kDa) | pI | Representative similarity to proteins in database | E value | Identity (positives) | GenBank  Accession  no. |
| --- | --- | --- | --- | --- | --- | --- | --- | --- | --- |
|  |  | Start | Stop |  |  |  |  |  |  |
| 1- | ATG | 16 162 | | 5.900 | 3.97 | Enterococcus phage IMEEF1 hypothetical protein | 4e-04 | 57% (71%) | AGR49007.1 |
| 2- | ATG | 224 499 | | 10.694 | 8.94 | Enterococcus phage IMEEF1 hypothetical protein | 6e-22 | 48% (67%) | AGR49014.1 |
| 3- | ATG | 589 816 | | 8.801 | 5.48 | Enterococcus phage ECP3 hypothetical protein | 7e-42 | 91% (94%) | YP_009147198.1 |
| 4- | ATG | 897 1106 | | 8.484 | 5.91 | Enterococcus phage BC611 hypothetical protein | 4e-39 | 91% (98%) | YP_006742215.1 |
| 5- | ATG | 1093 1527 | | 16.620 | 4.00 | Enterococcus phage IMEEF1 hypothetical protein | 5e-52 | 64% (76%) | AGR49009.1 |
| 6- | ATG | 1665 1799 | | 4.763 | 7.08 | Enterococcus phage IMEEF1 hypothetical protein | 1e-16 | 80% (86%) | AGR49010.1 |
| 7- | ATG | 1877 2251 | | 14.058 | 8.55 | Enterococcus phage IMEEF1 hypothetical protein | 3e-71 | 87% (93%) | AGR49011.1 |
| 8- | ATG | 2244 2630 | | 14.542 | 9.58 | Enterococcus phage IMEEF1 hypothetical protein | 1e-86 | 99% (100%) | AGR49012.1 |
| 9- | ATG | 2731 3054 | | 12.876 | 7.13 | Enterococcus phage IMEEF1 hypothetical protein | 5e-68 | 94% (96%) | AGR49013.1 |
| 10- | ATG | 3816 4121 | | 11.873 | 8.87 | Enterococcus phage vB_EfaS_IME198 hypothetical protein | 3e-51 | 78% (84%) | YP_009218949.1 |
| 11- | ATG | 4134 4439 | | 11.068 | 4.34 | Enterococcus phage IMEEF1 DNA binding protein | 4e-59 | 91% (93%) | AGR49015.1 |
| 12- | ATG | 4441 4707 | | 10.186 | 5.23 | Enterococcus phage IMEEF1 hypothetical protein | 1e-52 | 93% (94%) | AGR49016.1 |
| 13- | ATG | 4700 4816 | | 4.853 | 4.84 | Enterococcus phage IMEEF1 hypothetical protein | 9e-16 | 89% (94%) | AGR49017.1 |
| 14- | ATG | 4961 5170 | | 8.346 | 9.67 | Enterococcus phage IMEEF1 hypothetical protein | 5e-41 | 96% (98%) | AGR49018.1 |
| 15- | ATG | 5160 5378 | | 8.325 | 4.37 | Enterococcus phage IMEEF1 hypothetical protein | 2e-37 | 90% (91%) | AGR49019.1 |
| 16- | ATG | 5391 5603 | | 8.086 | 4.54 | Enterococcus phage IMEEF1 hypothetical protein | 5e-36 | 86% (94%) | AGR49020.1 |
| 17- | ATG | 5603 5863 | | 9.850 | 5.02 | Enterococcus phage BC611 hypothetical protein | 1e-52 | 94% (96%) | YP_006742208.1 |
| 18- | ATG | 5860 6054 | | 7.000 | 8.54 | Enterococcus phage BC611 hypothetical protein | 3e-36 | 100% (100%) | YP_006742207.1 |
| 19- | ATG | 6132 6551 | | 15.400 | 4.42 | Enterococcus phage IMEEF1 hypothetical protein | 6e-54 | 64% (78%) | AGR49023.1 |
| 20- | ATG | 6565 6765 | | 7.331 | 4.68 | Enterococcus phage BC611 hypothetical protein | 3e-38 | 95% (98%) | YP_006742206.1 |
| 21- | ATG | 6776 7363 | | 22.668 | 6.70 | Enterococcus phage IMEEF1 cytidine deaminase | 9e-98 | 79% (88%) | AGR49025.1 |
| 22- | ATG | 7386 7667 | | 11.035 | 9.18 | Enterococcus phage vB_EfaS_IME198 hypothetical protein | 8e-55 | 91% (93%) | YP_009218937.1 |
| 23- | ATG | 7680 7916 | | 9.259 | 3.72 | Enterococcus phage vB_EfaS_IME198 hypothetical protein | 4e-43 | 90% (94%) | YP_009218936.1 |
| 24- | ATG | 7900 8178 | | 10.279 | 4.48 | Enterococcus phage IMEEF1 hypothetical protein | 1e-40 | 88% (94%) | AGR49027.1 |
| 25- | ATG | 8237 8932 | | 26.533 | 8.09 | Enterococcus phage IMEEF1 ATP-dependent metalloprotease | 4e-130 | 77% (85%) | AGR49028.1 |
| 26- | ATG | 8925 9320 | | 15.178 | 4.11 | Enterococcus phage IMEEF1 hypothetical protein | 4e-81 | 89% (93%) | AGR49029.1 |
| 27- | ATG | 9322 9777 | | 17.484 | 4.27 | Enterococcus phage vB_EfaS_IME198 hypothetical protein | 1e-92 | 91% (93%) | YP_009218932.1 |
| 28- | ATG | 9852 12383 | | 95.749 | 6.47 | Enterococcus phage IMEEF1 DNA polymerase I | 3e-161 | 79% (87%) | AGR49034.1 |
| 29- | ATG | 12463 12648 | | 7.087 | 9.69 | Enterococcus phage BC611 hypothetical protein | 2e-33 | 93% (95%) | YP_006742205.1 |
| 30- | ATG | 12648 13001 | | 13.675 | 3.33 | Enterococcus phage IMEEF1 hypothetical protein | 2e-45 | 89% (98%) | AGR49036.1 |
| 31- | ATG | 13002 13214 | | 7.941 | 4.03 | Enterococcus phage IMEEF1 hypothetical protein | 4e-22 | 61% (78%) | AGR49037.1 |
| 32- | ATG | 13217 13426 | | 7.978 | 3.71 | Enterococcus phage IMEEF1 hypothetical protein | 2e-28 | 72% (89%) | AGR49038.1 |
| 33- | ATG | 13419 13646 | | 8.335 | 4.11 | Enterococcus phage IMEEF1 hypothetical protein | 7e-40 | 92% (93%) | AGR49039.1 |
| 34- | ATG | 13660 13854 | | 6.793 | 8.69 | Enterococcus phage IMEEF1 hypothetical protein | 2e-22 | 94% (96%) | AGR49040.1 |
| 35- | ATG | 13866 13982 | | 4.353 | 9.83 | Enterococcus phage BC611 hypothetical protein | 3e-11 | 89% (97%) | YP_006742200.1 |
| 36- | ATG | 13985 14548 | | 21.248 | 9.28 | Enterococcus phage IMEEF1 LPS glycosyltransferase | 7e-132 | 96% (98%) | AGR49044.1 |
| 37- | ATG | 14640 15272 | | 24.412 | 5.67 | Enterococcus phage IMEEF1 hypothetical protein | 1e-151 | 99% (100%) | AGR49045.1 |
| 38- | ATG | 15265 15834 | | 21.376 | 4.50 | Enterococcus phage IMEEF1 adenylate kinase | 4e-131 | 96% (98%) | AGR49046.1 |
| 39- | ATG | 15831 16136 | | 11.129 | 4.50 | Enterococcus phage IMEEF1 crossover junction endodeoxyribonuclease RuvC | 8e-66 | 99% (100%) | AGR49047.1 |
| 40- | ATG | 16402 16731 | | 12.402 | 4.14 | Enterococcus phage IMEEF1 hypothetical protein | 6e-72 | 95% (97%) | AGR49048.1 |
| 41- | ATG | 16731 17759 | | 39.677 | 6.36 | Enterococcus phage IMEEF1 hypothetical protein | 0 | 99% (100%) | AGR49049.1 |
| 42- | ATG | 17752 18192 | | 16.934 | 8.76 | Enterococcus phage IMEEF1 HNH homing endonuclease | 5e-101 | 96% (99%) | AGR49050.1 |
| 43- | ATG | 18263 18489 | | 8.838 | 8.36 | Enterococcus phage IMEEF1 hypothetical protein | 4e-44 | 99% (100%) | AGR49052.1 |
| 44- | ATG | 18455 19207 | | 28.658 | 6.77 | Streptococcus phage SPQS1 DNA methyltransferase | 0 | 99% (100%) | YP_008320519.1 |
| 45- | ATG | 19220 20584 | | 50.718 | 4.67 | Enterococcus phage IMEEF1 replicative DNA helicase | 0 | 99% (99%) | AGR49053.1 |
| 46- | ATG | 20596 21372 | | 29.639 | 8.46 | Enterococcus phage BC611 DNA replication protein | 0 | 99% (99%) | YP_006488761.1 |
| 47- | ATG | 21421 21774 | | 14.062 | 9.60 | Enterococcus phage IMEEF1 transcriptional regulator | 8e-80 | 98% (100%) | AGR49055.1 |
| 48- | ATG | 21849 22733 | | 34.031 | 5.20 | Enterococcus phage IMEEF1 DNA primase | 0 | 95% (97%) | AGR49056.1 |
| 49- | ATG | 22809 22997 | | 7.110 | 7.09 | Enterococcus phage BC611  hypothetical protein | 9e-38 | 98% (100%) | YP_006742197.1 |
| 50- | ATG | 23163 23414 | | 9.636 | 4.62 | Enterococcus phage IMEEF1 hypothetical protein | 4e-52 | 96% (96%) | AGR49060.1 |
| 51- | ATG | 23414 23788 | | 14.461 | 4.89 | Enterococcus phage IMEEF1 hypothetical protein | 1e-82 | 98% (99%) | AGR49061.1 |
| 52- | ATG | 23790 23936 | | 5.495 | 3.49 | Enterococcus phage BC611 host recBCD nuclease inhibitor | 2e-16 | 92% (92%) | YP_006742196.1 |
| 53- | ATG | 24726 24851 | | 4.847 | 10.62 | Enterococcus phage IMEEF1 hypothetical protein | 6e-14 | 90% (90%) | AGR49063.1 |
| 54- | ATG | 25351 25551 | | 7.405 | 9.23 | Enterococcus phage IMEEF1 hypothetical protein | 6e-38 | 91% (96%) | AGR49064.1 |
| 55- | ATG | 25553 26068 | | 19.802 | 9.47 | Enterococcus phage vB_EfaS_IME198 HNH homing endonuclease | 1e-34 | 43% (58%) | YP_009218914.1 |
| 56- | ATG | 26103 26489 | | 14.914 | 4.16 | Enterococcus phage IMEEF1 hypothetical protein | 2e-70 | 78% (90%) | AGR49065.1 |
| 57- | ATG | 26492 26926 | | 16.536 | 5.03 | Enterococcus phage IMEEF1 hypothetical protein | 2e-99 | 99% (99%) | AGR49066.1 |
| 58- | ATG | 26979 27803 | | 30.154 | 4.72 | Enterococcus phage IMEEF1 hypothetical protein | 0 | 99% (99%) | AGR49067.1 |
| 59+ | ATG | 28309 28533 | | 8.408 | 7.28 | Enterococcus phage vB_EfaS_IME198 HNH homing endonuclease | 7e-05 | 33% (60%) | YP_009218914.1 |
| 60+ | ATG | 28535 28963 | | 16.671 | 8.31 | Enterococcus phage IMEEF1 hypothetical protein | 7e-90 | 87% (95%) | AGR49069.1 |
| 61+ | GTG | 29083 29274 | | 7.253 | 7.09 | Enterococcus phage SAP6 head morphogenesis protein | 0 | 99% (99%) | AEM24734.1 |
| 62- | ATG | 29330 30040 | | 26.086 | 5.68 | Enterococcus phage VD13 the lysin protein | 6e-154 | 89% (93%) | YP_009036394.1 |
| 63- | TTG | 30155 30376 | | 8.302 | 4.83 | Enterococcus phage IMEEF1 hypothetical protein | 8e-22 | 55% (69%) | AGR49071.1 |
| 64- | ATG | 30389 33415 | | 111.820 | 4.55 | Enterococcus phage IMEEF1 minor structural protein | 0 | 91% (95%) | AGR49072.1 |
| 65- | ATG | 33428 37420 | | 152.188 | 5.18 | Enterococcus phage IMEEF1 tail fiber protein | 0 | 99% (99%) | AGR49073.1 |
| 66- | ATG | 37434 40319 | | 101.640 | 4.84 | Enterococcus phage IMEEF1 minor capsid protein | 0 | 98% (99%) | AGR49074.1 |
| 67- | ATG | 40332 40556 | | 8.754 | 4.81 | Enterococcus phage IMEEF1 hypothetical protein | 3e-44 | 96% (97%) | AGR49075.1 |
| 68- | ATG | 40567 41007 | | 17.079 | 7.15 | Enterococcus phage vB_EfaS_IME198 hypothetical protein | 1e-101 | 99% (100%) | YP_009218892.1 |
| 69- | ATG | 41150 41839 | | 24.658 | 4.44 | Enterococcus phage IMEEF1 major tail protein | 2e-165 | 99% (99%) | AGR49077.1 |
| 70- | ATG | 41860 42294 | | 16.499 | 6.14 | Bacillus subtilis tail protein | 4e-07 | 30% (54%) | WP_033884269.1 |
| 71- | ATG | 42307 42687 | | 13.973 | 5.78 | Enterococcus phage BC611 hypothetical protein A958_gp18 | 2e-85 | 98% (100%) | YP_006488745.1 |
| 72- | ATG | 42672 43049 | | 14.799 | 5.06 | Enterococcus phage IMEEF1 hypothetical protein | 2e-84 | 98% (100%) | AGR49080.1 |
| 73- | ATG | 43065 43469 | | 15.817 | 4.85 | Enterococcus phage VD13 head-tail connector family protein | 3e-93 | 99% (99%) | YP_009036383.1 |
| 74- | ATG | 43532 43969 | | 14.896 | 4.45 | Enterococcus phage IMEEF1 major tail protein | 2e-90 | 95% (96%) | AGR48984.1 |
| 75- | ATG | 44124 44930 | | 29.869 | 5.53 | Enterococcus phage vB_EfaS_IME198 major capsid protein | 0.0 | 100% (100%) | YP_009218885.1 |
| 76- | ATG | 44979 45647 | | 23.853 | 4.73 | Enterococcus phage vB_EfaS_IME198 hypothetical protein | 3e-157 | 100% (100%) | YP_009218884.1 |
| 77- | ATG | 45758 46513 | | 28.755 | 4.96 | Enterococcus phage SAP6 phage head morphogenesis protein | 0 | 99% (99%) | AEM24734.1 |
| 78- | ATG | 46525 48060 | | 58.09 | 5.43 | Enterococcus phage IMEEF1 portal protein | 0 | 99% (99%) | AGR48988.1 |
| 79- | ATG | 48117 48506 | | 14.957 | 9.55 | Enterococcus phage IMEEF1 terminase large subunit | 5e-87 | 99% (100%) | AGR48989.1 |
| 80- | ATG | 48908 49711 | | 31.384 | 9.14 | Enterococcus phage IMEEF1 terminase large subunit | 0 | 98% (99%) | AGR48989.1 |
| 81- | ATG | 49775 50023 | | 8.368 | 4.89 | Enterococcus phage BC611  Holin | 3e-46 | 99% (100%) | YP_006742191.1 |
| 82- | ATG | 50042 50386 | | 12.486 | 5.21 | Enterococcus phage BC611 hypothetical protein | 1e-75 | 100% (100%) | YP_006488734.2 |
| 83- | ATG | 50400 50999 | | 22.716 | 4.39 | Enterococcus phage BC611 terminase small subunit | 5e-144 | 99% (100%) | YP_006488733.1 |
| 84- | GTG | 51118 51240 | | 4.596 | 8.91 | Hypothetical protein |  |  |  |
| 85+ | ATG | 51280 51594 | | 12.045 | 4.83 | Enterococcus phage vB_EfaS_IME198 hypothetical protein | 3e-67 | 99% (100%) | YP_009218877.1 |
| 86+ | ATG | 51594 51851 | | 10.072 | 4.11 | Enterococcus phage IMEEF1 hypothetical protein | 2e-42 | 95% (98%) | AGR48994.1 |
| 87+ | ATG | 51833 52237 | | 15.194 | 4.38 | Weissella minor leucine--tRNA ligase | 9.7 | 39% (54%) | WP_057786824.1 |
| 88+ | ATG | 52239 52619 | | 14.720 | 4.00 | Enterococcus phage IMEEF1 methyltransferase | 8e-56 | 82% (92%) | AGR48996.1 |
| 89+ | ATG | 52713 52862 | | 5.723 | 9.32 | Enterococcus phage VD13 hypothetical protein X878_0006 | 5e-24 | 86% (89%) | YP_009036367.1 |
| 90+ | ATG | 52875 53135 | | 9.628 | 5.21 | Enterococcus MULTISPECIES: NrdH-redoxin | 5e-10 | 43% (60%) | WP_010745735.1 |
| 91+ | ATG | 53137 53439 | | 11.962 | 8.36 | Enterococcus phage IMEEF1 hypothetical protein | 1e-59 | 90% (93%) | AGR48999.1 |
| 92+ | ATG | 53459 53830 | | 13.603 | 4.03 | Erysipelotrichaceae bacterium I46 ABC transporter ATP-binding protein | 9.4 | 34% (50%) | WP_065530647.1 |
| 93+ | ATG | 54274 55032 | | 28.455 | 6.13 | Bacillus phage Shbh1 HNH homing endonuclease  Staphylococcus phage K putative HNH endonuclease  Lactococcus phage Phi4.2 HNH endonuclease | 1e-16  1e-13  2e-15 | 47% (69%)  41% (63%)  34% (51%) | YP_009275325.1  YP_009041365.1  ALM64160.1 |
| 94+ | ATG | 55107 55814 | | 27.629 | 5.01 | Enterococcus phage IMEEF1 hypothetical protein | 1e-57 | 57% (65%) | AGR49002.1 |
| 95+ | ATG | 55815 56033 | | 7.987 | 7.03 | Enterococcus phage IMEEF1 hypothetical protein | 5e-37 | 90% (91%) | AGR49003.1 |
| 96+ | ATG | 56035 56247 | | 7.919 | 9.13 | Enterococcus phage IMEEF1 hypothetical protein | 5e-38 | 96% (97%) | AGR49004.1 |
| 97+ | GTG | 56435 56575 | | 5.133 | 9.63 | Enterococcus phage BC611 hypothetical protein | 2e-24 | 98% (100%) | YP_006742186.1 |
| 98+ | ATG | 56653 56823 | | 6.304 | 9.86 | Bacillus phage Eldridge  Enterococcus phage SANTOR1 HNH homing endonuclease | 5e-07  1e-05 | 54% (66%)  51% (71%) | YP_009274804.1  YP_009284748.1 |
| 99+ | GTG | 56837 57166 | | 12.288 | 9.37 | Enterococcus phage vB_EfaS_IME198 HNH homing endonuclease | 4e-31 | 55% (67%) | YP_009218914.1 |
| 100+ | TTG | 58139 58261 | | 4.709 | 10.11 | Hypothetical protein |  |  |  |
| 101- | ATG | 58240 58542 | | 11.096 | 6.62 | Enterococcus phage IMEEF1 hypothetical protein | 1e-56 | 92% (95%) | AGR49006.1 |

*+, Right orientation; −, left orientation.
