# Supplementary material for: The Bacteriophage EF-P29 Efficiently Protects against Lethal Vancomycin-Resistant Enterococcus faecalis and Alleviates Gut Microbiota Imbalance in a Murine Bacteremia Model
Source: Front Microbiol. 2017 May 9;8:837. doi: 10.3389/fmicb.2017.00837 (PMC5423268; doi:10.3389/fmicb.2017.00837)
Supplement: Supplementary file 4 [file Table_4.DOC]

**Table S4. A global genome comparison of phages.**

|  | EF-P29 | IME198 | VD13 | IME-EF1 | SP-QS1 | BC-611 | SAP6 |
| --- | --- | --- | --- | --- | --- | --- | --- |
| G+C content | 39.77% | 40.02% | 40.00% | 40.04% | 39.87% | 40.45% | 40.00% |
| Genome size (bp) | 58,988 | 58,000 | 55,073 | 57.081 | 58.305 | 53.996 | 58.619 |
| Identity of EF-P29 | 100% | 97% | 94% | 94% | 93% | 95% | 94% |
| Query coverage of EF-P29 | 100% | 90% | 84% | 87% | 85% | 78% | 87% |
| GenBank no. | KY303907 | KT932699 | KJ094032 | KF192053 | HE962497 | AB712291 | JF731128 |
