# Supplementary figures and images for: The Bacteriophage EF-P29 Efficiently Protects against Lethal Vancomycin-Resistant Enterococcus faecalis and Alleviates Gut Microbiota Imbalance in a Murine Bacteremia Model
Source: Front Microbiol. 2017 May 9;8:837. doi: 10.3389/fmicb.2017.00837 (PMC5423268; doi:10.3389/fmicb.2017.00837)

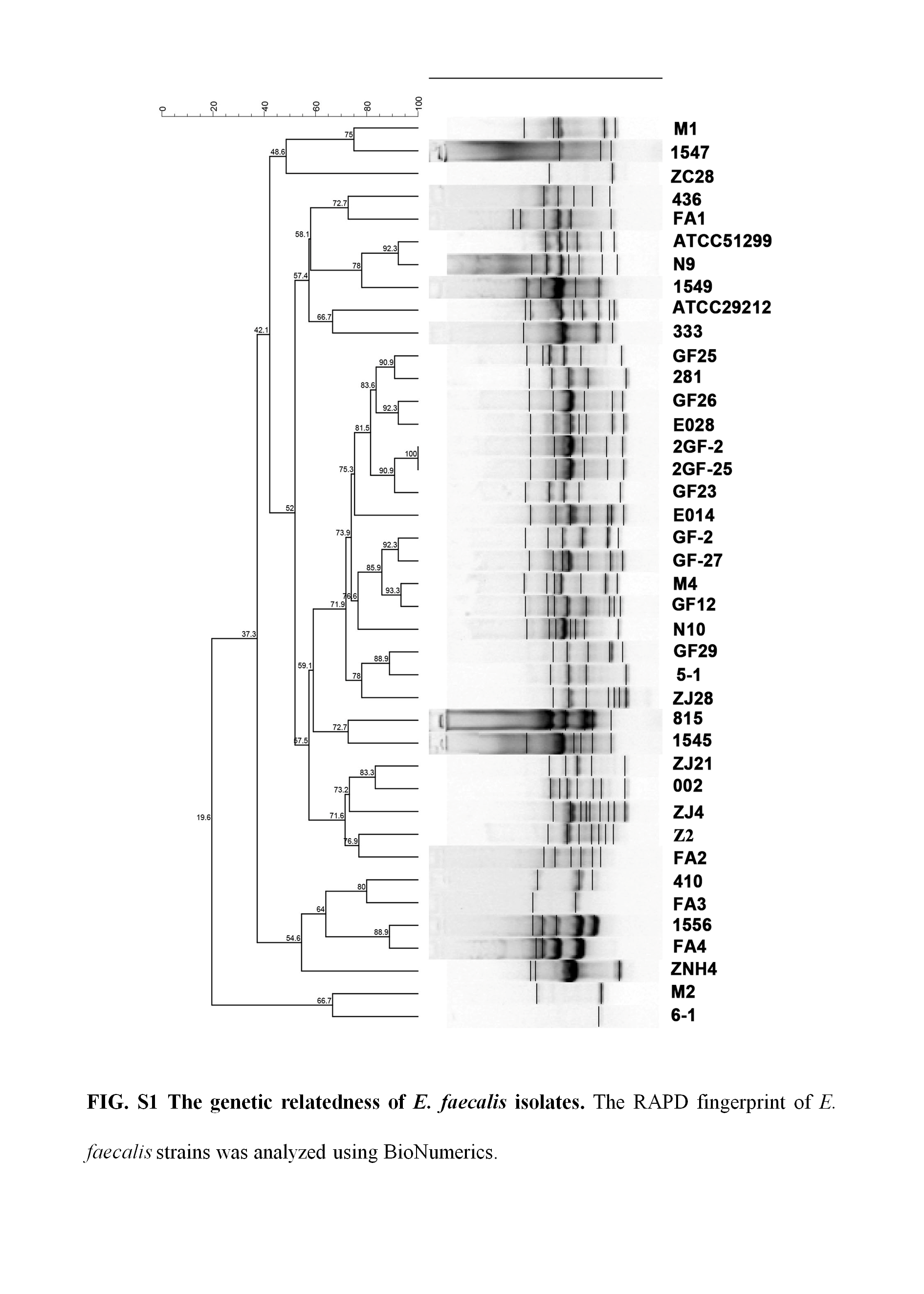

Supplement: Supplementary file 5 [file Image_1.TIF]

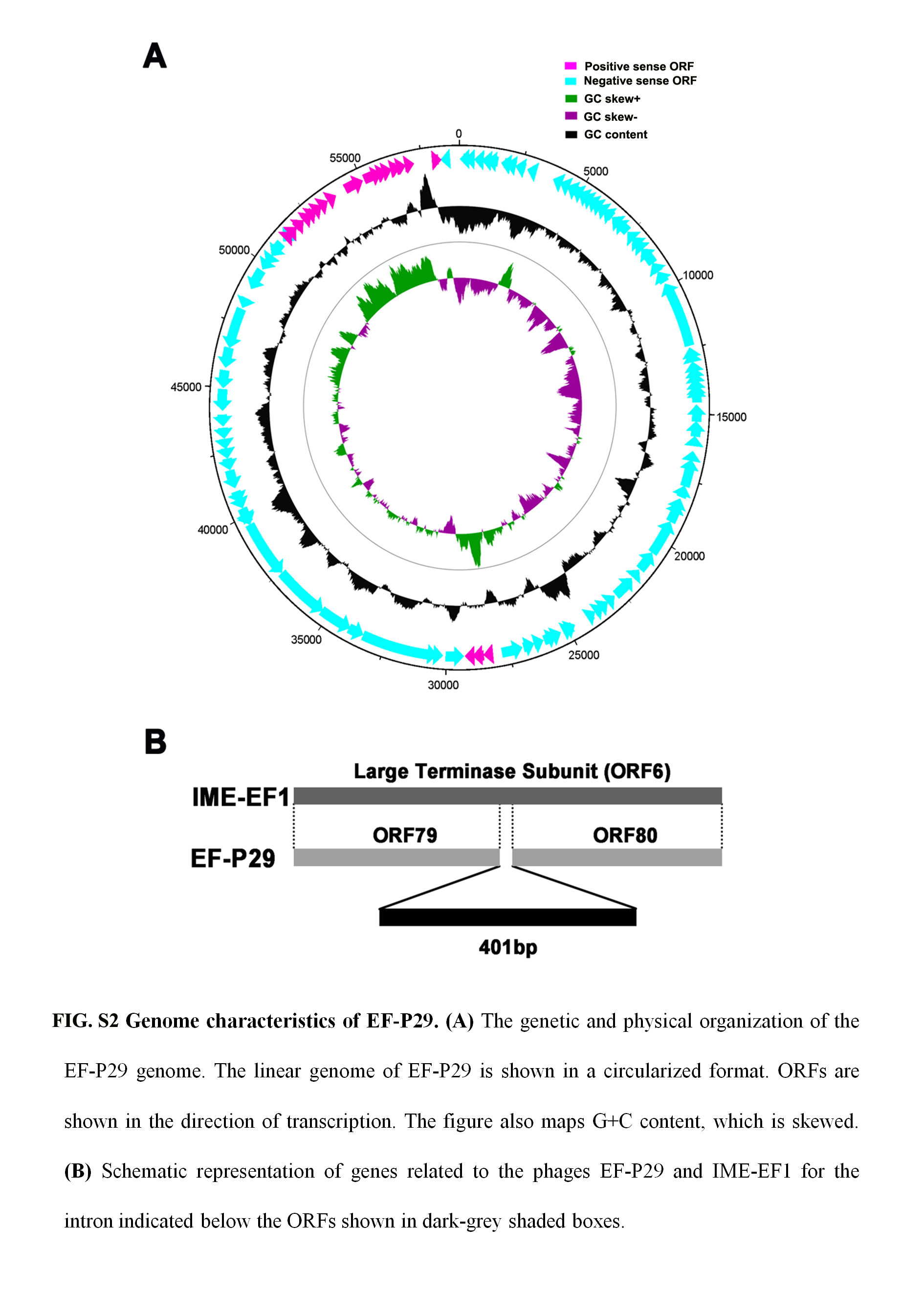

Supplement: Supplementary file 6 [file Image_2.TIF]

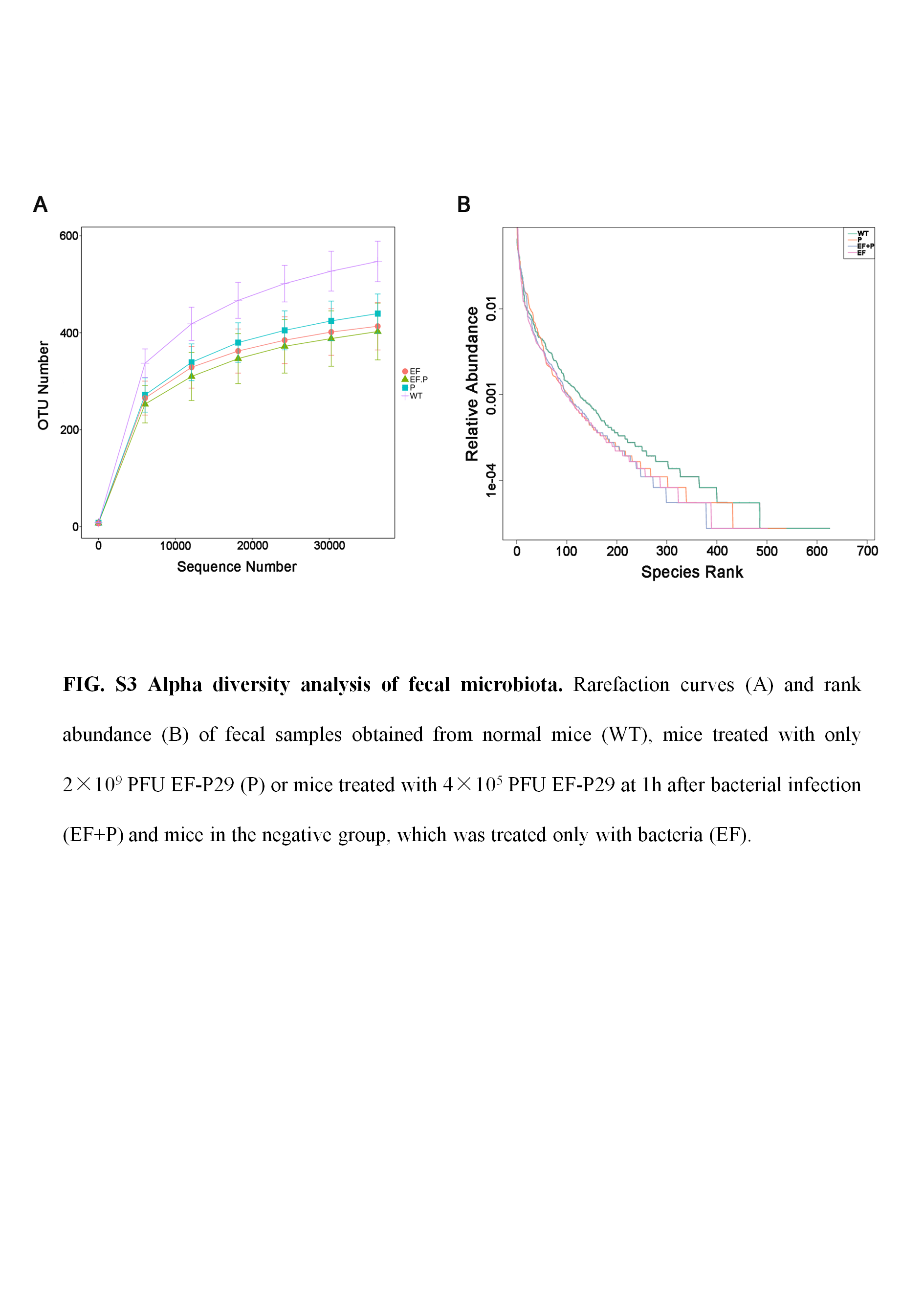

Supplement: Supplementary file 7 [file Image_3.TIF]

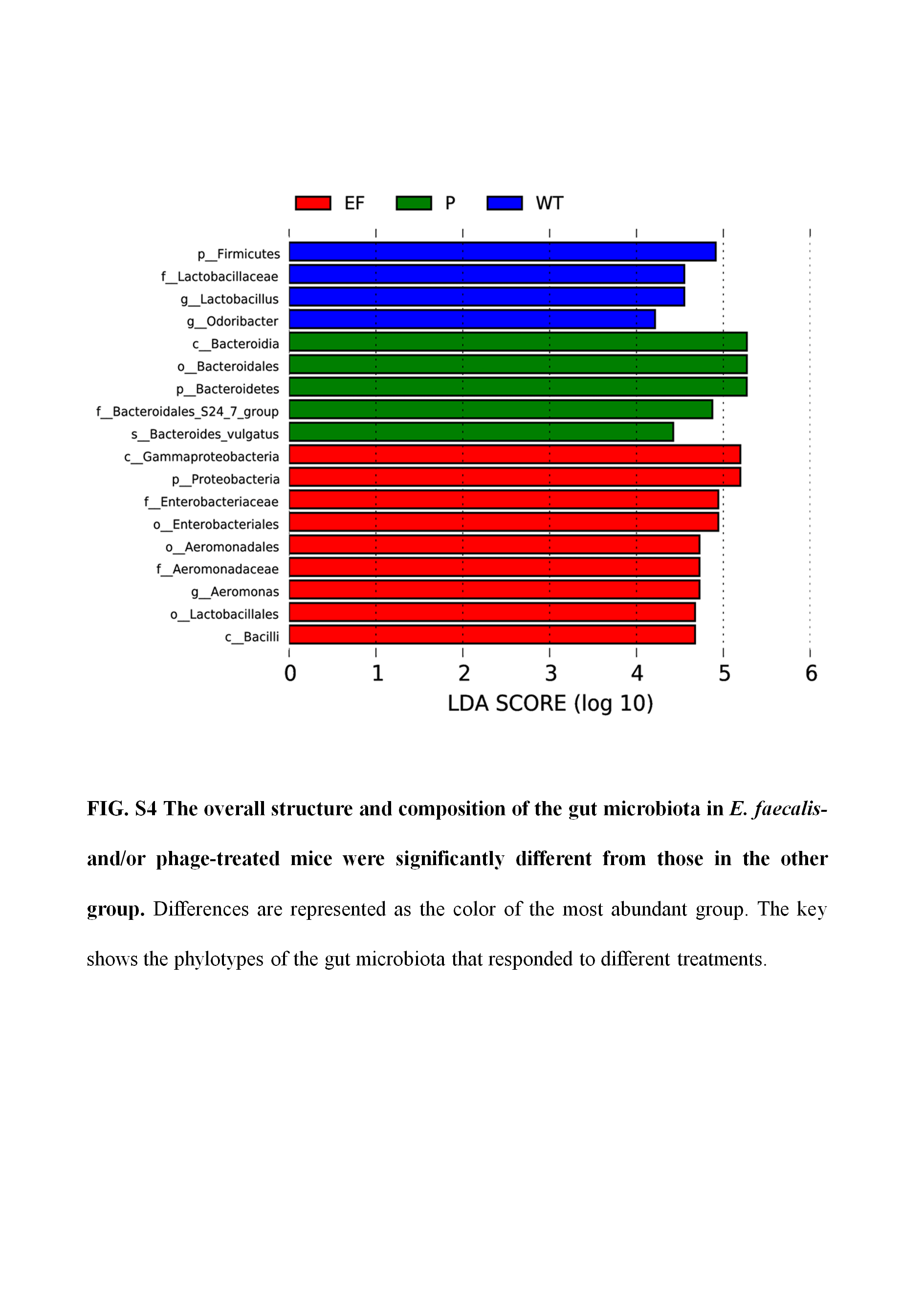

Supplement: Supplementary file 8 [file Image_4.TIF]

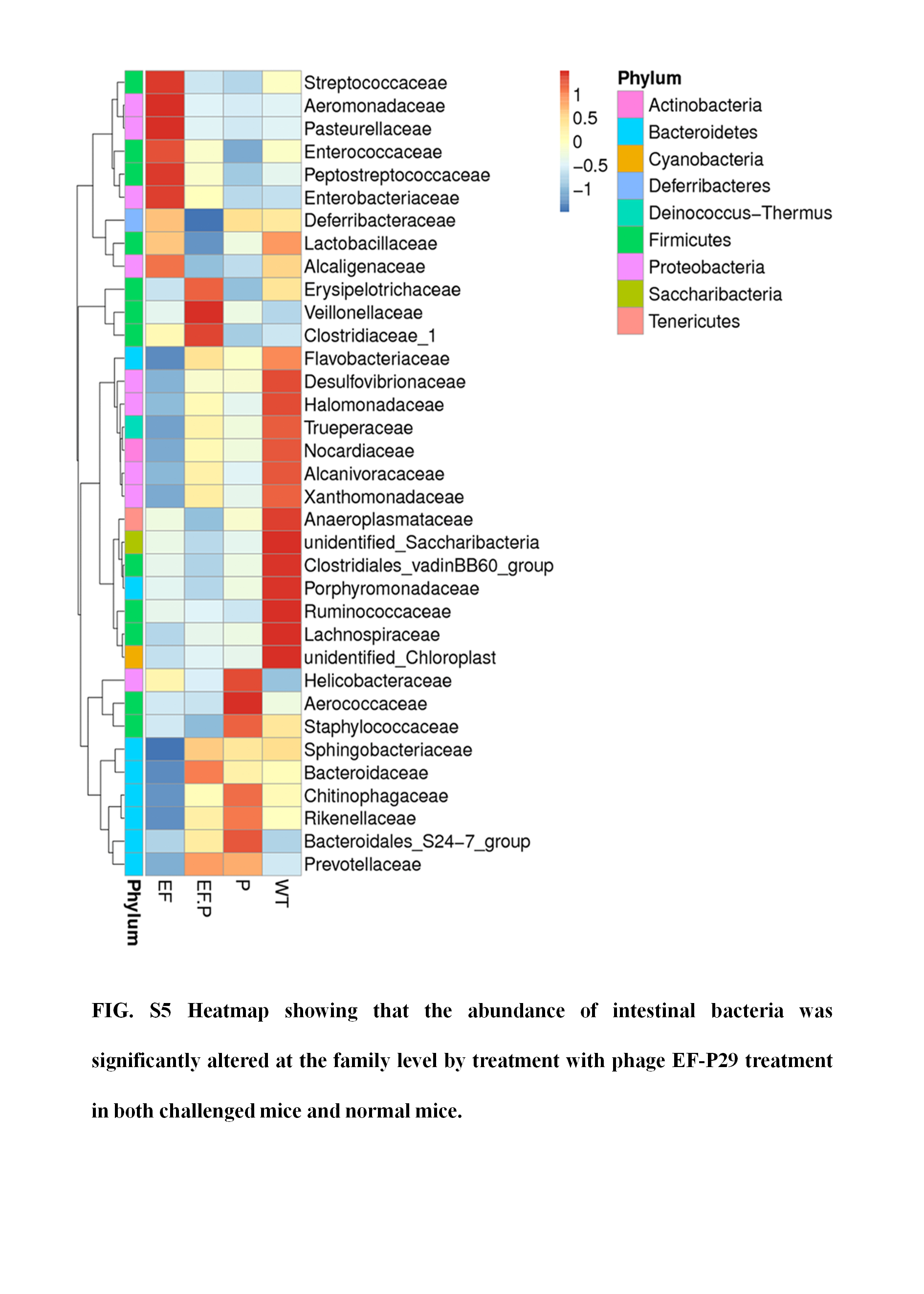

Supplement: Supplementary file 9 [file Image_5.TIF]
